# 293. Risk factors for COVID-19 associated pulmonary aspergillosis (CAPA) in severe COVID-19 and impact of airborne fungal contamination within negative pressure isolation room

**DOI:** 10.1093/ofid/ofac492.371

**Published:** 2022-12-15

**Authors:** Jung Ah Lee, Sanggwon Ahn, Se Ju Lee, Jinnam Kim, Ki Hyun Lee, Chang Hyup Kim, Joon-sup Yeom, Nam Su Ku, Su Jin Jeong, Jung Ho Kim, Jung Ho Hwang, Dongeun Yong, Jun Yong Choi, Jin Young Ahn

**Affiliations:** Yonsei University College of Medicine, Seoul, Seoul-t'ukpyolsi, Republic of Korea; Yonsei University, Seoul, Seoul-t'ukpyolsi, Republic of Korea; Yonsei University College of Medicine, Seoul, Seoul-t'ukpyolsi, Republic of Korea; Yonsei University College of Medicine, Seoul, Seoul-t'ukpyolsi, Republic of Korea; Yonsei University College of Medicine, Seoul, Seoul-t'ukpyolsi, Republic of Korea; Yonsei University College of Medicine, Seoul, Seoul-t'ukpyolsi, Republic of Korea; Division of Infectious Diseases, Department of Internal Medicine, Yonsei University College of Medicine, Seoul, Seoul-t'ukpyolsi, Republic of Korea; Division of Infectious Diseases, Department of Internal Medicine, Yonsei University College of Medicine, Seoul, Seoul-t'ukpyolsi, Republic of Korea; Yonsei University College of Medicine, Seoul, Seoul-t'ukpyolsi, Republic of Korea; Yonsei University College of Medicine, Seoul, Seoul-t'ukpyolsi, Republic of Korea; Yonsei University, Seoul, Seoul-t'ukpyolsi, Republic of Korea; Yonsei University College of Medicine, Seoul, Seoul-t'ukpyolsi, Republic of Korea; Yonsei University College of Medicine, Seoul, Seoul-t'ukpyolsi, Republic of Korea; Yonsei University College of Medicine, Seoul, Seoul-t'ukpyolsi, Republic of Korea

## Abstract

**Background:**

COVID-19 increase the risk of invasive pulmonary aspergillosis. However, the risk factors and fungal origin of COVID-19 associated pulmonary aspergillosis (CAPA) is not fully defined yet. We aim to identify the risk factors for CAPA in severe COVID-19 and evaluate association between fungal contamination within the air of negative pressure rooms and diagnosis of CAPAs.

**Methods:**

We performed a retrospective case-control study to identify risk factors for CAPA with 420 severe COVID-19 patients from March 2020 to January 2022 who admitted to a tertiary care hospital in South Korea. CAPA was defined with modified AspICU criteria. Control, matched by admission date and severity of COVID-19 at admission, was selected for each case. Air sampling and fungal culture was done on Jan 2022 with a microbial air sampler (MAS-100NT) at 11 spaces in the COVID-19 designated isolation ward including 9 negative pressure isolation rooms (IRs). A cross-sectional comparison between rooms with and without airborne fungal contamination was performed.

**Results:**

A total of 420 COVID-19 patients were hospitalized during the study period, and 51 patients were diagnosed with CAPA (prevalence 12.14%, incidence 6.26 per 1000 patient•day). Multivariate analysis showed that older age (odds ratio [OR] 1.051, 95% confidence intervals [CI] 1.006-1.009, p=0.025), mechanical ventilator use (OR 2.692, 95% CI 1.049-6.911, p=0.04), and lymphopenia (OR 4.353, 95% CI 1.727-10.975, p=0.02) were independent risk factors for CAPA. (Table 1, 2) Aspergillus spp. was identified within the air from 7 out of 11 spaces including 6 IRs and 1 doctors’ room. (Figure 1). All 6 IRs with positive aspergillus culture were being occupied by patients at least 8 days. Among 6 patients, 3 had already been diagnosed with CAPA whereas the other 3 were not diagnosed with CAPA through the observation period. Among 4 patients in isolation rooms without airborne aspergillus contamination, one patient had been diagnosed as CAPA before air sampling. (Table 3).

**Figure:**
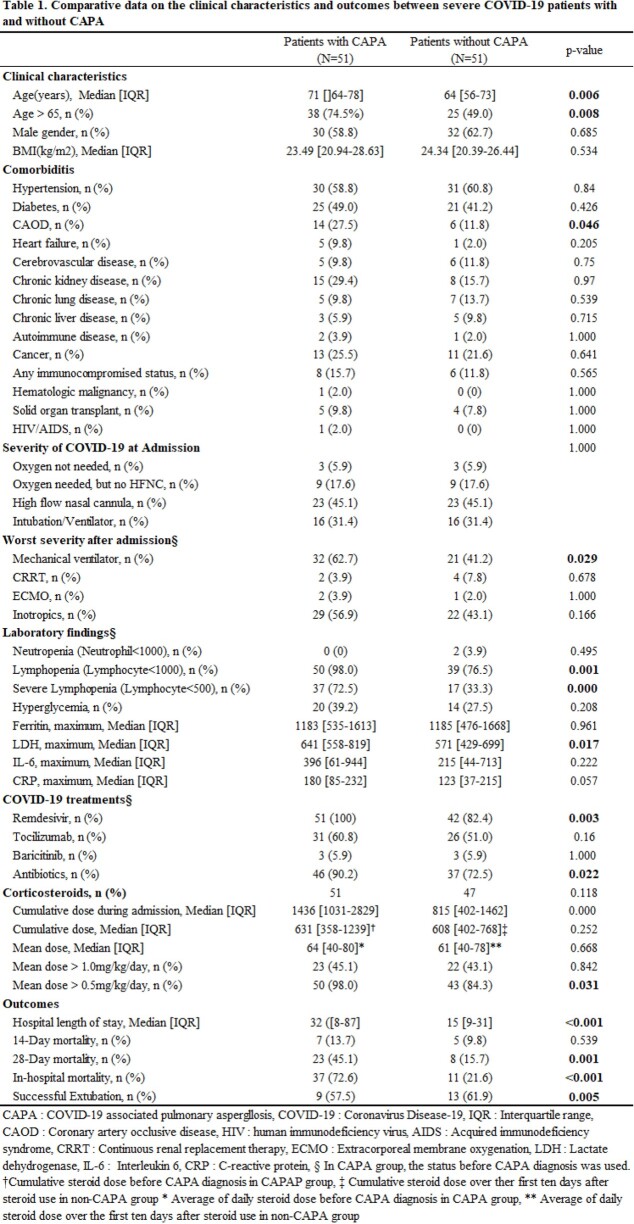

**Figure:**
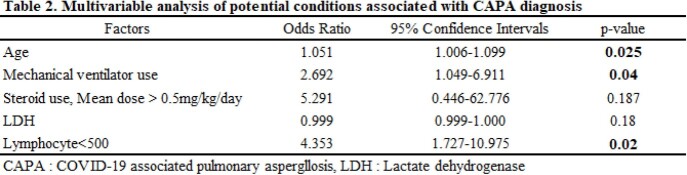

**Figure:**
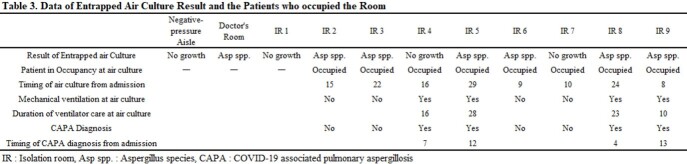

**Conclusion:**

Association between CAPA and airborne aspergillus contamination within the negative pressure room could not be demonstrated in this study. Rather than environmental factors, patient factors such as older age, ventilator care, and lymphopenia were found to be associated with CAPA diagnosis.

**Figure:**
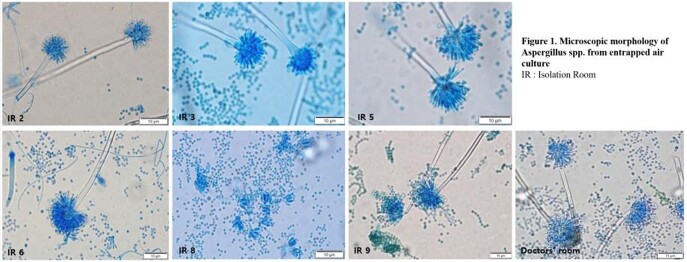

**Disclosures:**

**All Authors**: No reported disclosures.

